# Integrating Individual Animal and Population Welfare in Zoos and Aquariums

**DOI:** 10.3390/ani13101577

**Published:** 2023-05-09

**Authors:** Louis DiVincenti, Allen McDowell, Elizabeth S. Herrelko

**Affiliations:** 1 Independent Researcher, Hamlin, NY 14464, USA; 2 North Carolina Aquarium on Roanoke Island, P.O. Box 967, Manteo, NC 27954, USA; allen.mcdowell@ncaquariums.com; 3 Smithsonian’s National Zoo and Conservation Biology Institute, Washington, DC 20008, USA; herrelkoe@si.edu

**Keywords:** zoo animal welfare, aquarium, population welfare

## Abstract

**Simple Summary:**

The science of animal welfare can be approached along a continuum of perspectives. Historically, we considered animal welfare at a distance, through a big-picture examination of population-level parameters (e.g., longevity, reproductive success). In recent decades, scientists and practitioners have advanced the field and optimized animal welfare by incorporating a focused approach examining each individual (e.g., their lived experiences). Population-level welfare evaluations are key to validating parameters used to measure individual animal welfare and have an important role when individual animal welfare cannot be easily measured. However, there are also situations in which individual and population welfare may be in conflict, and managers must consider maximizing population welfare at the expense of individuals. We examine these cases and explore opportunities for the integration of individual and population-level welfare to promote optimal well-being for animals in zoos and aquariums.

**Abstract:**

Over the last 50 years, animal welfare science has advanced dramatically, especially in zoos and aquariums. A shifting focus from population-level welfare parameters such as reproductive success and longevity (macroscopic, big-picture concepts) to the subjective experience of individual animals (microscopic, focused concepts) has led to more effective animal welfare assessments and improvements in animal welfare. The interplay between individual animal and population welfare for captive animals is critical to the way zoos and aquariums operate to realize their welfare and conservation missions, especially when these missions conflict with one another. In this report, we explore the intersection of individual animal and population welfare in zoos and aquariums and how these two concepts may support one another or be in conflict.

## 1. Introduction

Societal interest in animal welfare is not new and has guided the humane care of animals across the spectrum of settings in which humans use animals, from food production to exhibition for educational and recreational purposes. Furthermore, this interest has spurred animal caregivers to scientifically evaluate the welfare implications of those practices. Historically, reproductive success measured at the population level, often in terms of production (e.g., numbers of offspring, gallons of milk, dozens of eggs, pounds of meat, etc.), was considered the key indicator of animal welfare [1]. When populations were producing large numbers of offspring, the welfare of the individuals within that population was assumed to be good. Over time, this emphasis on population-based outputs led to intensive “factory farm” practices in which the welfare of individual animals was not routinely evaluated. Ruth Harrison’s 1964 exposé *Animal Machines* revealed the reality of these practices and demonstrated that population-based parameters do not adequately protect animal welfare [2]. The subsequent public outcry prompted the formation of what became known as the Brambell Committee and the basis of the modern approach to animal welfare, the Five Freedoms [3]. The Five Freedoms’ assertion that welfare includes the physical and mental states of an individual animal and that animals have a right to specific minimal levels of care was revolutionary at the time [4]. Over the last 50 years since the Brambell Committee, the field of animal welfare science has advanced dramatically from merely measuring population-level production parameters to minimize negative welfare to closely evaluating how an individual animal responds to its environment to promote positive welfare [5]. This shift has been accompanied by accrediting bodies articulating animal welfare principles and guidelines for zoos and aquariums centered on individual animals. The World Association of Zoos and Aquariums (WAZA) defines animal welfare as “a state that is specific for every individual animal; is how the animal experiences its own world and life through its association with pleasant experiences specific for that species such as vitality, affection, safety and excitement, or unpleasant experiences such as pain, hunger, fear, boredom, loneliness, and frustration” [6]. From this definition and building on the Five Freedoms, the Five Domains have emerged as a more recent model to assess animal welfare with particular emphasis on preventing compromises in welfare across a wide range of general needs animals have [7]. This modern concept of animal welfare represents a microscopic approach, focusing on the experiences of individual animals, not just groups or populations of animals residing at a facility, and using animal-based indicators such as behavior to assess welfare. With this definition, zoos and aquariums have made a concerted effort to optimize animal welfare through research, advanced veterinary care, exhibit modifications, behavioral management and enrichment programs, staff education and training, and other approaches [8,9]. Despite this focus on individual animal welfare, zoos and aquariums are responsible for managing populations of animals, both within their own institutions and throughout the larger community of accredited facilities around the world. Accordingly, caregivers must also consider population welfare when making animal management decisions.

Although welfare measured at the population level historically focused on production outputs without necessarily understanding the cost to the individuals, a modern concept of population welfare that specifically accounts for the well-being of individuals within that population has recently emerged. In contrast to WAZA’s definition of animal welfare focused on individual animals, population welfare has been defined as “coherence between the adapted needs of a species with critical social and environmental resources” [10]. In other words, population welfare is connected to species conservation and population health and maximized through ensuring a species has an environment that matches its needs. It represents a macroscopic approach to animal welfare centered on the optimal environment for a population to thrive. ‘Group welfare’ has become a widely used term within zoos and aquariums. For the purposes of this paper, we use the term population welfare to refer to the collective physical, behavioral, and psychological well-being of groups of animals housed within zoos and aquariums as well as welfare assessments that utilize indicators measured at the group rather than the individual level. For example, population welfare may refer to the well-being of large groups of fish and invertebrates in aquarium exhibits, antelope housed in extensive enclosures, or other highly social, inter-dependent species such as nonhuman primates, meerkats, and others, and considers the shared needs of the individuals as a species or a group. In this way, population welfare correlates with the conservation mission of accredited zoos and aquariums as they seek to maintain healthy populations of various species not only for continued public display but also as a hedge against extinction in the wild. However, this conservation mission can result in competing interests when individual and population welfare goals are not aligned.

**Figure 1:**
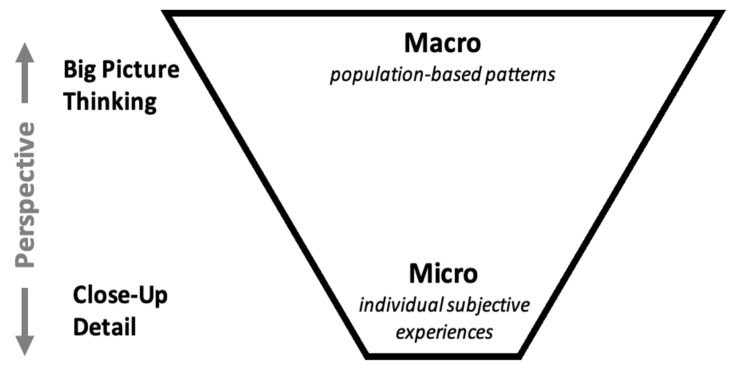
Spectrum of animal welfare approaches from the macro (big-picture thinking) concept typically focused on population-based patterns to the micro (close-up detail) concept typically focused on an individual’s lived experiences.

The dichotomy of individual (microscopic) and population (macroscopic) approaches to animal welfare has been an important challenge in formulating meaningful welfare assessments. The critical components of an animal’s welfare, their lived experiences (e.g., the Five Domains, the Five Opportunities to Thrive; see [4,11], respectively), are nested within the big-picture concepts typically used in population welfare assessments (Figure 1). As we will discuss, such big-picture concepts can be applied to individuals to gather information about their welfare, but the fine detail of individual animal welfare assessments is difficult to apply to large populations without significant resources. This difficulty has prevented the application of welfare methods developed for individual animals in zoos and aquariums to not only wild animals in their natural habitats (i.e., in situ environments) but also to members of some species within zoos and aquariums. The experimental nature of many approaches to animal welfare assessments, such as intervention effectiveness testing, makes those approaches inherently challenging to apply in environments where animal intervention is avoided and/or confounding variables cannot always be controlled. Subsequently, in situ animal welfare research tends to be theoretical in nature [12]. While the welfare of wild animals is certainly connected to conservation and the mission of the zoos and aquariums, an examination of the population welfare of wildlife is beyond the scope of this paper. The interplay between individual animal and population welfare for captive animals is critical to the way zoos and aquariums operate to realize both their welfare and conservation missions. In this report, we explore the intersection of individual animal and population welfare in zoos and aquariums and how these two concepts may support one another or be in conflict. 

## 2. Synthesizing Individual and Population Welfare

Zoos and aquariums have always informally assessed the welfare of the animals in their care. Typically, a population-level approach was integrated into the perception of the welfare of individual animals. Early attempts at more formal welfare assessments often focused on “inputs,” or animal care and husbandry parameters provided to the animals. This approach assumed that animals given an optimal environment would experience optimal welfare. Indeed, the development and promulgation of standards at the species (or higher) level has been one important method to systematically improve animal welfare in zoos and aquariums. To this end, the Association of Zoos and Aquariums (AZA)’s Animal Welfare Committee participates in the production of Animal Care Manuals that establish best practices in many areas of animal care and management, including environmental parameters, exhibit design, transport, social environment, nutrition, veterinary care, reproduction, behavior management, and research with the goal of maximizing “welfare potential”, the potential that individual animals will experience good welfare based on the care they receive [9]. Similarly, the European Association of Zoos and Aquariums (EAZA) produces best practice guidelines through various Taxon Advisory Groups with the goal of merging expert husbandry knowledge and making it widely available [13]. While these guidelines correctly recognize that environmental and population-level factors (i.e., inputs) have a large impact on individual animal welfare, such best practices assume all individuals of a species or taxon share a core set of common needs. Yet, because animal welfare is fine-tuned at an individual level, adherence to these guidelines does not guarantee that all individuals within that population will have good or even adequate welfare. The growing study of individual differences highlights the impact of personality and lived experiences on an animal’s ability to cope and thrive within a given environment, from groups of gorillas responding differently to varying visitor volume [14] to the influence rank has on the enriching effect of training sessions [15] and the importance of challenges being appropriate for an individual’s ability and resources [16]. These studies highlight the idea that individuals in the same setting can and do experience various states of welfare, and consideration of individual differences within a group is critical to understanding the welfare of each individual.

Beyond the fact that not all individuals of a species have identical needs, the lack of species-specific information regarding the physical and psychological needs of many species is an additional challenge to the use of taxon-level guidelines [17]. Despite the increased interest in animal welfare across all species and the promotion of animal welfare research over the last two decades, there remains a paucity of validated welfare indicators for many species maintained by zoos and aquariums. This is especially true in understudied taxa such as reptiles, amphibians, fish, and invertebrates whose specific natural histories and physical, nutritional, social, and psychological needs may not even be well understood. To address this issue, accredited zoos and aquariums have prioritized research utilizing the individuals in their care to characterize animal needs and key indicators of welfare (e.g., [18]). For these species, welfare assessments may be performed macroscopically simply because we lack the knowledge of individual welfare needs to accurately assess it at the individual level at this time.

Although a critical need, identifying and validating physiological, behavioral, and psychological indicators associated with animal welfare requires significant time and expense. As discussed, animal welfare research in zoos and aquariums is particularly challenging because strict experimental conditions cannot be imposed in most real-world situations, and the relatively small numbers of each species housed at a single facility means that generalizing any findings to individuals at other facilities is complicated. For this reason, studies which combine macro- and micro-level approaches to welfare assessments can be critical to identifying and validating welfare indicators, thus improving individual animal welfare. Furthermore, the multidimensional nature of animal welfare requires assessments to be based on scientific knowledge that considers many aspects including provision of resources, caregiver interactions, positive and negative affective states and events, behavior, and others [19]. To this end, the Elephant Welfare Initiative (EWI) was a large-scale, epidemiologic study of elephants in North American zoos that documented the prevalence of positive and negative welfare states in individuals across facilities. The study included nearly 300 elephants in over 70 facilities and used a population-level, epidemiologic approach to determine the environmental, management, and husbandry factors that could impact elephant welfare [20]. The EWI’s population-level findings could then be used to assess and improve the welfare of individual animals. For example, the size of an elephant exhibit was a common resource-based, input-type metric used to assess elephant welfare prior to the EWI. However, the EWI found that exhibit size was not correlated with better foot or musculoskeletal health [21] or reduced stereotypic behaviors [22], which are commonly accepted animal-based indicators of elephant welfare. In other words, results from the EWI suggested that moving an individual elephant displaying stereotypical behavior to a larger exhibit would, as a single intervention, be unlikely to improve that animal’s welfare. In contrast, the EWI found that spending more time in larger social groups was protective against performance of stereotypic behaviors [22], providing caregivers with a science-based intervention to improve the welfare of an elephant displaying stereotypic behavior. As essentially a large-scale epidemiology study, the major challenge of the EWI was that it was limited to elephant care facilities as they existed at the time, thus specific hypotheses for improving elephant welfare could not be tested. Despite this limitation, the EWI generated hypotheses for targeted research to test interventions likely to improve welfare in the future. Given the challenges of studying animal welfare with the small populations and multiple confounding variables found in zoos and aquariums, this type of evidence-based approach is not possible without population-level welfare research.

Population-level welfare research of this nature may be even more impactful assisting zoos and aquariums in selecting species most likely to experience good welfare in captivity. The importance of selecting species well adapted for an institution’s physical and climatic environment has been discussed for decades [23]. However, considering specific information regarding the needs of individuals of different species housed in captivity could lead to a paradigm shift in approaches to collection plans, facilities’ master plans, and day-to-day operations of zoos and aquariums. For example, using species-typical behaviors, such as natural hunting behavior, general activity levels, ranging, and territorial patrolling, Clubb and Mason [24] were able to explain the signs of poor welfare (e.g., abnormal behavior and poor reproductive success) seen in some captive carnivore species but not others. As a result, Clubb and Mason make recommendations regarding species likely to experience good welfare in zoos and aquariums and species that should perhaps be avoided [24]. At the very least, population-level research can inform enclosure designs to facilitate improved welfare for the animals who will live there. More importantly, this approach to animal welfare science relies on identifying trends which will likely yield the best results for individuals of a given species or taxon group, but recognizes that individual assessments, when possible, are still critical to forming a complete picture of animal’s welfare in a given setting. 

While population-level research can guide welfare assessments of individuals, population-level approaches cannot replace animal-based assessments at the micro level, which require direct observation of individual animals [25]. To capture individual differences in the subjective experience of welfare, assessors must have knowledge of and familiarity with an animal as an individual. At the most basic level, this familiarity requires the ability to individually identify each animal in the group. However, in large aquariums in which potentially hundreds of individuals of the same species, with a nearly identical appearance, live in the same enclosure, it may not be possible for caregivers to identify animals as individuals, much less be familiar with an individual’s behavior and temperament, in order to assess the welfare of individuals. A similar challenge might exist in an expansive enclosure in which large herds of hoof stock are managed more extensively than traditional zoos. It is important to note that animals that can be individually identified, even when housed in large enclosures or large groups, should be assessed individually whenever possible. Facilities may choose to house animals in these more naturalistic ways to improve welfare, but these settings complicate individual animal welfare assessments and can make population-level assessments more appropriate. 

How can facilities meaningfully assess welfare in these situations? Group observations make welfare assessments possible when individual assessments are not feasible, and they are effective when used appropriately [26,27]. Even in population-level analyses, the behavior of the individual is important to note as an individual that looks or acts differently is a potential indicator of a welfare concern. These outliers should be a stimulus for investigation and may warrant subsequent individual assessments to determine the cause of the outlier behavior and the possible welfare implications, for both the individual and the group. For many of the species in which group observations may be utilized, the welfare of each individual is so intricately tied to the other animals in the group that population-level welfare assessments may be more meaningful than individual assessments. For example, many fish spend their whole life as a member of a group, and this schooling behavior has extremely high biological significance affecting a wide variety of adaptive functions [28]. Aquatic invertebrates such as coral and bryozoans that form colonies and are literally connected to other members of the group represent another important example. More complex examples involve animals such as some sea anemones that reproduce by budding and species such as the Mexican topminnow (*Poeciliopsis 2 monacha-lucida*), an all-female fish species that reproduces by cloning with all individuals sharing the same genotype for thousands of years [29]. Population-level indices such as fecundity, adult survivorship, neonatal mortality, and stereotypic behavior may be useful to indicate general issues with animal well-being within these groups that require further characterization at the individual level. However, it is important to recognize that these parameters are largely affected by negative welfare states. In other words, animals experiencing negative welfare are, in general, likely to have decreased fecundity and adult survivorship and increased neonatal mortality and stereotypic behaviors [30]. Animals experiencing a minimum level of welfare are likely to be free from diseases, injury, and malnutrition, and these factors clearly affect longevity, reproductive success, and other parameters that can be assessed at the population level [17]. A more forward-looking approach to welfare assessments focused on optimization of welfare incorporates indicators of positive welfare such as animal autonomy, play behaviors, positive human–animal relationships, and social interactions [31]. Such positive welfare indicators require validation at the population level to be useful. For population-level welfare assessments to be effective in promoting optimal welfare, the monitoring schedule must be frequent enough with a low enough threshold for intervention to identify specific problems before they cause significant welfare effects [32]. 

## 3. Tension between Individual and Population Welfare

As we have discussed, although the experience of individual animals is critical, population-level considerations can be important tools in the assessment and optimization of animal welfare. Given the historical use of population-level indicators that may compromise individual animal welfare, it is unsurprising that tension between population and individual animal welfare occurs in zoos and aquariums. Importantly, different concepts of animal welfare among caregivers and other stakeholders may be the root of this tension in many cases [33]. For example, Veasey [34] found that veterinary staff prioritized health-centered parameters such as ease of preventive and emergency care and morbidity and mortality rates over a more holistic approach to welfare assessments that included elements relating to biologic functioning, natural living, and affective states of individual animals when assessing animal welfare. Emphasis on certain aspects of welfare over others is expected as a result of the different roles caregivers may have within an institution and the nuanced nature of animal welfare. Achieving consensus through a team approach to evaluations of animal welfare is critical to relieving some tension that occurs as a result of the lack of consensus on how to prioritize different aspects of animal welfare. For this reason, many accredited zoos and aquariums have developed objective scoring systems that require input from a range of stakeholders including veterinarians and animal caregivers to help foster a collaborative approach and to facilitate decision making [34].

Conflicts also arise when there are differences in defining and measuring welfare among stakeholders. For example, animal caregivers and even members of the public may form a bond with an individual animal and prioritize the welfare of that individual, whereas veterinarians and administrators charged with managing populations of animals may take a more macroscopic approach to welfare. These conflicts arise because humans have such a profound impact on animals, and humans are increasingly interested in active animal management to mitigate this impact [35]. Obviously, prioritizing the well-being of individual animals best minimizes suffering and maximizes positive welfare states in individuals. However, when caregivers prioritize population welfare, they adopt a broader approach to animal stewardship that emphasizes species preservation as a greater good. Both approaches have merit, and both have their limitations. When possible, the use of scientific knowledge to make animal welfare decisions is critical to at least fostering collaboration among all stakeholders when complete conflict resolution is not possible. However, animal welfare science often relies on tacit ethical judgements and assumptions on what matters in humans’ interactions with animals [36]. In reality, ethical decision-making regarding animal welfare issues is complex, and when science cannot resolve conflicts, animal professionals must use a reasoned approach to animal ethics to communicate their concerns and find a collaborative way to move forward. 

Although some conflicts may arise from differing concepts of animal welfare, legitimate challenges in which caregivers must weigh the welfare of individuals against the welfare of the population do occur. As we discuss these situations, it is important to note again that animal welfare is individual but decisions regarding individuals have potential consequences for the welfare of other animals in the group and inclusion of those consequences in evaluations of management decisions for an individual is inevitable. Challenges to individual welfare as a result of group dynamics in social species is perhaps the most common situation animal managers need to consider. In the wild, social species develop a hierarchy in which dominant animals require some form of submission from subordinate animals. The negative effects on the welfare of subordinates outweigh the positive effects these animals realize as members of the group (e.g., protection from predators). However, in a zoo and aquarium setting where animals are spatially restricted, subordinates may not have sufficient opportunity to escape from aggression or resources guarded by dominants. Caregivers must recognize the natural history of species, accepting some level of natural aggression towards subordinates, and that intervention to protect these lower ranking individuals may actually cause more deleterious effects on the welfare of the group. In some cases, an individual within a population may have special needs that compromise the welfare of other individuals, especially in social species. For example, in the author’s experience, a baboon with a significant health problem that requires frequent veterinary care may lead to disruptions within the troop, such as displaced aggression on subordinate troop members. Non-target individuals may experience fear, distress, and physical injury when the target individual is captured, removed from, and/or re-introduced to the troop for treatments or other interventions (e.g., [37]). This fear and distress can complicate future efforts to provide treatment to those individuals when they experience a health concern. In other cases, when a social animal requires close observation in the hospital, animal care staff may strategically select a healthy companion as a social partner. The situation inherently decreases the healthy companion’s opportunity for optimal welfare in the short term, but drastically increases the likelihood of a better welfare experience for the animal under veterinary care. In these cases, animal care teams need to explicitly clarify what values and objectives are being prioritized (e.g., individual vs. group welfare) so that all stakeholders understand how and why management decisions were made.

Additionally, resources may be limited in zoos and aquariums such that additional expenses for the welfare of an individual animal may preclude other interventions that may provide a benefit to more individuals. Geriatric animals, especially solitary animals that require relatively large exhibits such as tigers, represent another challenge to zoo resources. To meet their conservation goal, zoos need to exhibit reproductively viable animals, but a commitment to lifelong welfare requires facilities to invest in resources to maintain post-reproductive animals. In other cases, an exhibit designed specifically to encourage species-specific natural behaviors could challenge an individual’s welfare as the individual ages. For example, an orangutan exhibit designed to promote species-typical climbing behaviors may need to be altered for a geriatric individual who develops arthritis. While intended to improve the welfare of the geriatric individual, these alterations could lead to the other orangutans in the enclosure spending more time on the ground and an atypical behavior profile for the species. In these situations, caregivers are required to weigh the benefit to one individual against the potential harm to other individuals in the group. 

Individual animal welfare is contingent on effective population planning, both within and among institutions. Although animal welfare is a core principle of modern zoos and aquariums, their core purpose is conservation [38]. Historically, conservation and animal welfare have occasionally been at odds, as management for conservation often involves activities that may be detrimental to individual animals such as killing of invasive species to promote an indigenous endangered species [39]. In these situations, staff within zoos and aquariums often revert to arguments concerning the benefits to wildlife or the species concerned generally. While important from a utilitarian point of view, these arguments are irrelevant to individual animal welfare. More recently, the term “compassionate conservation” has been coined to encourage those concerned primarily with conservation and those concerned primarily with animal welfare ethics to work together based on a shared commitment to nature (see [40]). There are opportunities for conservationists and animal welfare ethicists to come together. Ultimately, animal welfare ethics differs from the concept of animal rights in that interventions that may be harmful to an individual, such as euthanasia or confinement, are acceptable, provided the individual does not experience “unnecessary pain and suffering” and the resulting benefits outweigh the cost of the suffering. In these cases, conservationists and animal welfare ethicists can agree on a more utilitarian approach to animal welfare to weigh the cost to the individual with the greater good (e.g., long-term viability) the population will realize as a result. 

Animals in zoos and aquariums are managed intensively with the goal of population conservation. Successful breeding in zoos and aquariums is essential to the long-term conservation of species in captivity, and, more importantly, as a hedge against extinction in the wild. Inevitably, cooperative management programs must balance the goal of conserving a population with the welfare of individual animals when considering transfer, introduction, breeding, and contraception decisions. A transfer between facilities may be the most stressful experience in a captive animal’s life. For example, experiencing an inter-zoo transfer was identified as a risk factor for elephant mortality in zoos in one study [41]. Yet, the transfer of genetic material among institutions is the key component essential for population viability. Alternative approaches to inter-zoo transfers, such as using positive reinforcement to train animals for semen collection and artificial insemination, can improve individual animal welfare by reducing the need for transfers and also achieve the goal of long-term population viability. While perhaps a refinement, even this approach to breeding introduces challenges to individual animal welfare. Perhaps most importantly, denying animals a natural mating opportunity prevents expression of a key species-specific behavior its wild counterparts are highly motivated to perform. Secondly, development of successful artificial insemination procedures requires research, and, in many cases, trial and error. The individuals who participate in these procedures may experience anesthesia, hormone therapy, and other interventions that may have negative effects on their welfare, at least in the short-term. Yet, the greater good of these procedures will ultimately lead to both enhanced individual animal welfare through reduction of the stress of transport and improved population viability through more efficient breeding.

With respect to animal acquisition and breeding, aquariums face unique challenges in terms of balancing individual animal and population welfare, as well as their conservation missions. Removing animals from the wild for display in aquariums challenges the welfare of the individuals and, depending on the methods used for capture, threatens the long-term viability of wild populations of many species (see [42] for a more thorough review of these issues). For this reason, the development of culturing techniques for many species has been a priority for accredited aquariums, and recent successes have led to the potential for large-scale acquisition of animals through these captive breeding programs. Eliminating the stress animals experience from capture, transport, and introduction to an aquarium enclosure is a significant improvement for both individual animal welfare and conservation of wild populations. Cultured animals also tend to acclimatize better to human care, and, since these individuals typically present a lesser health risk to established aquarium populations than wild-caught individuals, they may be able to avoid some quarantine procedures and medical treatments that have historically been stressful [43]. Captive fish breeding, though, requires the same considerations for individual and population welfare as other species previously discussed.

The ultimate example of compromising individual animal welfare for the greater good of the population is the use of culling “surplus” individuals as a management tool. From a conservation genetics standpoint, it is likely that zoos and aquariums need to produce more offspring than there are existing spaces to ensure long-term population viability [44]. Contraception and other methods used in zoos and aquariums to delay and/or reduce the frequency of breeding have long-term negative effects on fertility in many species and may be associated with deleterious health conditions in treated individuals [45]. Furthermore, physical isolation of animals to prevent breeding may have negative welfare effects on those individuals, especially in social species. In these cases, achievement of optimal welfare for individuals, and populations, may require natural breeding based on a species’ life history, which can result in populations too large for existing facilities. These concerns have led some managers within the zoo and aquarium field to consider euthanasia of healthy individuals as a necessary management tool. While an in-depth discussion of the ethics of management euthanasia, or culling, is beyond the scope of this paper, both AZA and WAZA recognize that humane euthanasia is a tool for managing the demographics, genetics, and diversity of animal populations within zoos and aquariums [46]. For the purposes of this discussion, it assumed that culling methods are consistent with the principles of humane euthanasia, which requires that methods to induce the most rapid, painless, and distress-free death possible are utilized [47]. When an individual is euthanized, or culled, for management reasons, it may be argued that that individual’s welfare is compromised for the good of the population [48]. However, others argue that euthanasia is welfare neutral when performed humanely since it results in the death of the animal, and thus no subjective experience of a positive or negative state [49]. This dichotomy reflects a fundamental difference in stakeholder values. Although an in-depth discussion of the level of self-awareness required for euthanasia to be considered welfare negative is beyond the scope of this paper, the distinction is important when considering the range of species housed in zoos and aquariums that may be affected by management euthanasia. 

While general aspects of management euthanasia are applicable to both terrestrial and aquatic species, aquarists and invertebrate caregivers are faced with unique considerations when discussing culling as a population management tool. While attempts are made to treat aquatic animals with chronic parasitic or other infectious diseases, culling affected individuals is often a necessary tool if other treatments have failed. Aquarists justify this management approach with a focus on group health and welfare. The cost to individual health and welfare is accepted because infectious diseases are often so virulent within enclosed aquatic habitats that a focus on individual welfare will come at a cost to the group in terms of the potential for other animals to become affected. As discussed above, breeding programs in aquariums are increasing to improve individual animal welfare and minimize stress on wild populations. However, in the authors’ experience, as reproductive rates increase, there is also an increase in juveniles with deformities such as missing fins, misshapen bodies, and underdeveloped swim bladders. These conditions are deleterious to both individual animal and population welfare, and, while controversial, aquarists do utilize euthanasia as a management tool in these cases. Additionally, the management of surplus animals becomes problematic since it is nearly impossible to breed a specific number of animals. Culling these excess individuals, and at what point in the fish development process, to prevent overcrowding and poor welfare is another controversial topic in this growing field. 

## 4. Conclusions

The field of zoo and aquarium animal welfare science has advanced dramatically over the last 50 years from merely evaluating population-level indicators such as reproductive success to assessing the welfare of individual animals using a variety of parameters indicating negative and positive welfare states. Often, population-level observations and epidemiological research have led to the identification and validation of welfare indicators at the individual level. These population-level assessments have facilitated the development of best practices for the care of individual species and taxa and improvements in individual animal welfare, and, in some cases, make welfare assessments possible for large numbers of animals in zoos and aquariums. Ignoring population-level welfare and assessments limits the advancements of welfare science on an individual level. However, animal welfare is based on an individual animal’s subjective experience. Zoos and aquariums must balance individual animal and population welfare especially regarding breeding programs and management of geriatric and special-needs animals. 

Despite the advancements in animal welfare science through significant research investigating population and individual welfare, as discussed in this paper, much more work is needed to continue this progress. Population-level studies to validate indicators of welfare for individual animals are essential, especially for under-studied taxa such as invertebrates, fish, amphibians, and reptiles. Approaches to assessing the welfare of large populations, especially when individuals within a group have no identifying characteristics, that do not rely on production-based indicators (e.g., reproductive output) need to be developed and validated. Finally, research to understand individual differences within a species is needed to evaluate and improve best practice guidelines used to care for animals and facilitate animal welfare improvements.

## Data Availability

No new data were created for this manuscript.
